# Drink and Heredity

**Published:** 1900-08-18

**Authors:** 


### DRINK AND HEREDITY.

The Society for the Study of Inebriety is anxious to obtain evidence as to the true relation between inebriety and heredity. They are fully satisfied that drunken parents tend to have children who become drunkards. That seems to be granted. It is, however, on the further question, whether a parent who himself inherits no special tendency to inebriety can as a result of mere intemperate habits entail upon his children a potential inebriety, that they most earnestly desire to obtain evidence. The president of the society (Dr. Wynn Westcott, 396, Camden Road, N.) will be glad to receive communications on the subject. The problem is a thorny one, bristling with difficulties, not the least of which lies in the fact that many people will be glad to throw upon their parents the blame of their own misdoings, and that many, again, would rather attribute their weakness to the lapse of a single precedent generation than admit that they are sprung of tainted or neuropathic ancestry.

